# White matter microstructure disruption associated with PET and cognitive impairment in Alzheimer’s disease

**DOI:** 10.1371/journal.pone.0346661

**Published:** 2026-04-08

**Authors:** Marlon Gonzales, Xiaojian Kang, Christian Cort, Maheen M. Adamson, Steven Z. Chao, Byung C. Yoon, for the Alzheimer’s Disease Neuroimaging Initiative

**Affiliations:** 1 Meharry Medical College, Nashville, Tennessee, United States of America; 2 WOMEN CoE, VA Palo Alto Healthcare System, Palo Alto, California, United States of America; 3 Rehabilitation Service, VA Palo Alto Healthcare System, Palo Alto, California, United States of America; 4 United States Navy, Port Hueneme, California, United States of America; 5 Department of Neurosurgery, Stanford University School of Medicine, Stanford, California, United States of America; 6 Neurology Service, VA Palo Alto Healthcare System, Palo Alto, California, United States of America; 7 Department of Neurology, Stanford University School of Medicine, Stanford, California, United States of America; 8 Radiology Service, VA Palo Alto Healthcare System, Palo Alto, California, United States of America; 9 Department of Radiology, Neuroradiology Division, Stanford University School of Medicine, Stanford, California, United States of America; Nathan S Kline Institute, UNITED STATES OF AMERICA

## Abstract

Alzheimer’s disease (AD) is associated with regional brain atrophy as well as elevated positron emission tomography (PET) markers of amyloid-beta (e.g., [¹⁸F]florbetapir (FBP)) and tau protein (e.g., [¹⁸F]flortaucipir (FTP). White matter microstructures have also been shown to be disrupted in AD, but there is limited understanding of their specific associations with FBP, FTP, and cognitive impairment. Herein, we used both voxel-based and fixel-based analyses of diffusion tensor imaging (DTI) to characterize microstructural white matter changes associated with PET and cognitive changes in AD. A retrospective study was performed using the data from 381 ADNI-3 participants (F:M = 200:181). FBP and FTP results were correlated with DTI metrics, including the apparent fiber density (AFD), complexity (CX), functional anisotropy (FA), fixel number (FN), and mean diffusivity (MD). Linear regression analysis was performed, adjusted for age, sex, education, and cognitive impairment. Greatest negative correlations were observed between FN and FBP standardized uptake value ratio (SUVR) in 15 out of 18 white matter tracts examined (beta coefficients of −0.3991 to −0.2877). No significant correlation was observed between DTI measures and FTP SUVR, independently. However, combined PET positivity (FBP + /FTP+) generally showed the greatest reductions in CX, FN, and FA of various tracts, compared to single PET-positive or PET-negative groups. Widespread changes in FA were positively associated with cognitive impairment, with stronger associations seen with the Montreal Cognitive Assessment (MoCA) than the Mini-Mental State Examination (MMSE). Only females showed significant correlations between MD and FBP/FTP levels and showed more widespread correlations between FA and MD changes and cognitive impairment. Taken together, these findings suggest specific patterns of white matter microstructure disruption in AD with underlying sex differences and support their potential role as early biomarkers.

## 1. Introduction

Alzheimer’s disease (AD) is a neurodegenerative disorder characterized by progressive cognitive impairment. Approximately 6.9 million individuals aged 65 years and older are affected by AD in the United States, with the prevalence projected to rise to 13.8 million by 2060 [1]. AD is associated with abnormal accumulation of amyloid-beta plaques and neurofibrillary tangles that contain tau proteins [2]. Much effort has been made for earlier and more accurate diagnosis of AD, especially with the advent of anti-amyloid therapies for the treatment of early AD [3]. For instance, various positron emission tomography (PET) tracers such as [¹⁸F]florbetapir (FBP) and [¹⁸F]flortaucipir (FTP) that bind to amyloid-beta and tau proteins, respectively, are used for a more quantitative assessment of brain abnormalities associated with AD [4–6]. Magnetic resonance imaging (MRI) is also used to evaluate for regional brain atrophy associated with AD and to exclude other mimics of AD, but its role is limited in ruling in the AD diagnosis, especially at the early preliclinical stage [7,8].

Advances in MR techniques, including diffusion tensor imaging (DTI), have allowed for more microscopic assessment of the brain structures that would otherwise not be feasible with the conventional MRI. Based on the diffusion of water molecules along the direction of white matter fibers, DTI allows for the assessment of disruption in microscopic white matter structures in various neurological disorders [9]. Commonly used, voxel-based analysis metrics such as fractional anisotropy (FA) and mean diffusivity (MD) reflect the degree of tract directionality and total rate of diffusion, respectively [10–12]. Studies have shown that alterations in white matter microstructures along the AD continuum may potentially serve as early biomarkers. For instance, decreased FA and increased MD were observed in AD patients in the frontal, temporal, and parietal white matter as well as the corpus callosum in AD patients, compared to healthy controls [13]. Other studies have found significant correlations between FA and cognitive impairment [14,15]. The combination of hippocampal volume and hippocampal MD was shown to improve the diagnostic accuracy of AD [16]. Studies examining the correlations between the DTI metrics and elevations in amyloid-beta and tau proteins have been more conflicting, however. Some of the results suggest that there is a significant correlation between elevated tau and MD, but not amyloid-beta [17], while others have shown significant correlations between DTI metrics, including FA and MD, with elevated amyloid-beta [18].

One of the shortcomings of voxel-based analysis is that while they reflect general changes to the white matter structure, they are limited in their resolution of multiple fiber populations within a single voxel and in characterizing specific types of white matter microstructural changes [9,11,19]. More recently, advances in diffusion MRI models led to the development of strategies to study individual fiber population within a voxel, known as “fixel” [11]. The fixel-based analysis allows for more detailed evaluation of various fiber characteristics, including the density of the nerve fibers (apparent fixel density; AFD), number of major fibers (fixel number; FN), and complexity of fiber bundles within a voxel (complexity; CX). Utilizing the fixel-based analysis, prior studies have identified significant reduction in fiber density of patients with AD in various tracts, compared to those with mild cognitive impairment and controls [20]. Fixel-based-analysis metrics were also shown to be decreased in the cingulum bundle, uncinate fasciculus, and anterior thalamic radiations in amyloid-beta-positive individuals with mild cognitive impairment compared to control in the Swedish BioFINDER-2 study [19]. Furthermore, the same study revealed a decrease in fixel-based-analysis metrics were correlated with memory decline [19].

Sex differences in AD and underlying pathophysiology are also an active area of research. There is a notable difference in the prevalence of AD between the sexes with nearly two-thirds of Americans with AD being females, and 11% of females and 9% of males aged 65 years and older reported to have AD [1]. Studies have shown that amyloid-beta positive females show greater tau-PET retention than amyloid-beta positive males, primarily in the temporal lobe [21]. In addition, a study using the OASIS-3 database showed that females with AD had lower free-water-corrected FA compared to males with AD [22]. However, our understanding of the sex differences in DTI changes associated with AD remains limited.

The aim of this study is to further investigate specific DTI changes that are directly associated with the amyloid-beta and tau levels using both voxel-based and fixel-based analyses and using a relatively large cohort of 381 right-handed participants from the Alzheimer’s Disease Neuroimaging Initiative (ADNI). Furthermore, we sought to determine whether DTI metrics are more disrupted in individuals who have an elevation in both amyloid-beta and tau, and whether there are sex differences in the type and degree of DTI changes. Lastly, we explored the potential role of DTI metrics as imaging markers in supplementing the assessment of cognitive impairment. We hypothesized that the combination of voxel-based and fixel-based analyses would confer a more sensitive evaluation of white matter changes associated with elevated amyloid-beta and/or tau, and that individuals with elevation in both amyloid-beta and tau would demonstrate more pronounced changes in DTI metrics. We also hypothesized that females and males have distinctive DTI changes in association with elevated amyloid-beta and tau, as well as with cognitive impairment.

## 2. Methods

### 2.1. Participants

Data used in the preparation of this article were obtained from the Alzheimer’s Disease Neuroimaging Initiative database (adni.loni.usc.edu) [23]. The ADNI was launched in 2003 as a public-private partnership, led by Principal Investigator Michael W. Weiner, MD. The primary goal of ADNI has been to test whether serial magnetic resonance imaging, positron emission tomography, other biological markers, and clinical and neuropsychological assessment can be combined to measure the progression of mild cognitive impairment and early AD. For up-to-date information, see www.adni-info.org. Demographic variables included age, sex, and years of education. Baseline cognitive function was assessed using Mini-Mental State Examination (MMSE) and the Montreal Cognitive Assessment (MoCA). Informed consent specific to this study was not necessary as this study only included data from a publicly available, de-identified database, ADNI.

### 2.2. PET imaging and classification

Baseline standardized uptake value ratio (SUVR) values of FBP and FTP were collected from ADNI-3. FBP positivity was defined as SUVR > 1.1 based on the prior study using the ADNI data [24]. FTP positivity was calculated based on past studies [25–27]. For each participant, regional FTP SUVR values were averaged across all available brain regions. The FTP SUVR cut-off was calculated as two standard deviations above the mean FTP SUVR of cognitively normal and FBP- individuals. A cut-off value of 1.265326 was determined, above which was classified as FTP + .

### 2.3. MRI acquisition and DTI analysis

All the imaging data used in the study were obtained from 3T scanners from GE, Siemens, and Philips. Standardized ADNI-3 protocols were used, although the exact sequence parameters may vary based on system hardware and software. DTI data from the baseline scan were used in this study with approximate sequence parameters as follows: T_R_ = 3300 ms, T_E_ = 71 ms, b = 500, 1000 and 2000 s/mm^2^, 112 total non-linear directions, voxel size = 2 × 2 × 2 mm^3^. More details of the MRI parameters can be found at https://adni.loni.usc.edu/help-faqs/adni-documentation/.

For DTI analysis, a similar workflow was utilized as previously reported [9]. Briefly, all the DTI metrics were obtained using the software package Mrtrix3 (https://www.mrtrix.org/) [28]. Mean diffusion metrics were subsequently calculated and compared between PET groups for all the fiber tracts after they were aligned and resampled to the International Consortium of Brain Mapping (ICBM) space [29,30] using SPM25 (https://www.fil.ion.ucl.ac.uk/spm/) in MATLAB (https://www.mathworks.com/, version 2024b). The white matter tracts and corresponding abbreviations are listed in S1 Table.

DTI images and analysis suffer from variation of the technical specifications across imaging sites and scanners [31]. Analysis of variance (ANOVA) was performed to examine the variability in DTI metrics among different MRI scanners (General Electric (GE), Philips, and Siemens 3T scanners; Matlab2024b). No significant differences were found for FA, MD, and AFD, while significant differences in CX and FN were seen between the scanners. Therefore, all the analyses were adjusted for the scanner types. As an additional note, no significant differences were found for the cortical thickness (*p* = .08) and TIV volume (*p* = .5) measurements.

### 2.4. Statistical analysis

Statistical analyses were performed using Matlab2024b and RStudio 4.4.3. Linear regression models were used to evaluate associations between DTI metrics, FBP/FTP SUVR, MMSE, and MoCA. All the regression analyses were adjusted for age, sex, education, and MRI scanner types. Comparisons between DTI metrics and FBP/FTP findings (Tables 1, 2, and S2, S3 Tables) were additionally adjusted for cognitive impairment status (e.g., cognitive normal, early mild cognitive impairment, late mild cognitive impairment, significant memory concern, and AD). For comparisons across the four FBP/FTP groups, ANOVA was used with a Bonferroni-adjusted *P* value for multiple comparisons (*p* < .05/4). For comparisons of FBP + /FTP + , FBP + /FTP-, or FBP-/FTP+ group against the FBP-/FTP- group (Table 2), the differences between DTI metrics between the PET-positive (A) and FBP-/FTP- (B) groups were calculated using the following formula: (A-B)/ mean (A,B) * 100. The significance level was set at *p* < .05. The Benjamini-Hochberg algorithm was performed for false discovery rate correction for multiple comparisons within all ROIs [32]. Sex-stratified analyses were performed for associations between DTI and FBP/FTP findings (S2 and S3 Tables), and between DTI and cognitive measures (S4 and S5 Tables), with models performed separately for males and females and sex omitted as a covariate.

**Table 1:** Correlations between FBP SUVR and DTI *(p <* .05 only).

Metric	Fiber_Tract	Β-Coefficient	R2	*p-*value
Complexity	CCF	−0.0658	0.1207	0.0015
	ATRL	−0.0611	0.1167	0.0030
	CSTL	−0.0582	0.1072	0.0035
	ILFL	−0.0534	0.0960	0.0068
	ATRR	−0.0541	0.1123	0.0083
	IFOL	−0.0479	0.1139	0.0123
	ILFR	−0.0479	0.0871	0.0159
	IFOR	−0.0462	0.1118	0.0210
	SLFBR	−0.0473	0.0923	0.0216
	SLFBL	−0.0446	0.0943	0.0303
	UNCR	−0.0497	0.1016	0.0336
	CSTR	−0.0409	0.1136	0.0349
	CgUL	−0.0476	0.0836	0.0392
	UNCL	−0.0416	0.1109	0.0467
Fixel Number	ATRL	−0.3991	0.0850	0.0032
	ATRR	−0.3949	0.0880	0.0039
	IFOL	−0.3941	0.0964	0.0041
	ILFL	−0.3979	0.0894	0.0045
	CCF	−0.3780	0.0980	0.0059
	CSTL	−0.3410	0.0801	0.0071
	SLFBL	−0.3888	0.1005	0.0101
	ILFR	−0.3534	0.0880	0.0110
	SLFBR	−0.3670	0.0932	0.0135
	CSTR	−0.3003	0.0853	0.0140
	IFOR	−0.3381	0.0978	0.0148
	CgUL	−0.3464	0.0768	0.0180
	UNCL	−0.3164	0.0846	0.0259
	UNCR	−0.3159	0.0863	0.0318
	CCO	−0.2877	0.0895	0.0431
Fractional Anisotropy	CSTR	−0.0480	0.1012	0.0006
	ATRR	−0.0455	0.1448	0.0008
	CgLR	−0.0443	0.1010	0.0032
	IFOR	−0.0407	0.1515	0.0034
	CSTL	−0.0384	0.0895	0.0072
	ILFR	−0.0354	0.1229	0.0110
	IFOL	−0.0352	0.1341	0.0117
	CCO	−0.0375	0.1582	0.0176
	CgLL	−0.0339	0.0913	0.0192
	UNCL	−0.0321	0.0982	0.0220
	ATRL	−0.0327	0.1377	0.0264
	SLFBR	−0.0308	0.0798	0.0304
	SLFBL	−0.0282	0.0741	0.0334
	CCF	−0.0280	0.1429	0.0336
	UNCR	−0.0304	0.0878	0.0338
	ILFL	−0.0269	0.1159	0.0475
Mean Diffusivity	CSTL	0.1105	0.1165	0.0296

**Table 2:** Group-wise comparisons between DTI metrics and PET positivity (<.0125 only).

Metric	Fiber Tract	R2	*p*-value	FBP- FTP- (n = 206)	FBP + FTP- (n = 132)	FBP- FTP+ (n = 5)	FBP + FTP+ (n = 38)
Complexity	ATRL	0.0310	0.0055	0.5764	−5.2%	6.1%	−7.7%
	CCF	0.0300	0.0063	0.5503	−5.0%	3.1%	−9.2%
	CSTR	0.0290	0.0076	0.4659	−4.6%	7.8%	−10.1%
Fixel Number	ILFL	0.0290	0.0078	2.9814	−6.0%	−1.2%	−11.6%
	SLFBL	0.0290	0.0081	3.0653	−6.3%	−4.0%	−12.1%
	ATRL	0.0290	0.0092	2.8983	−5.9%	2.2%	−11.1%
	IFOL	0.0280	0.0107	2.8597	−5.7%	−1.1%	−11.8%
	SLFBR	0.0270	0.0124	3.0070	−6.1%	−3.7%	−11.6%
Fractional Anisotropy	CgLR	0.0460	0.0003	0.2678	−8.6%	−10.2%	−17.7%
	ATRR	0.0420	0.0006	0.3026	−8.2%	−16.9%	−10.5%
	CSTR	0.0430	0.0007	0.4085	−6.5%	−3.6%	−8.1%
	IFOR	0.0370	0.0014	0.3439	−7.2%	−7.5%	−9.4%
	CSTL	0.0360	0.0027	0.4151	−6.1%	−3.5%	−7.2%
	ILFR	0.0340	0.0029	0.3400	−6.3%	−4.8%	−10.3%
	IFOL	0.0330	0.0035	0.3465	−6.7%	−5.1%	−9.1%
	ATRL	0.0320	0.0037	0.3141	−7.0%	−6.2%	−11.7%
	CCO	0.0280	0.0069	0.3427	−6.1%	−2.6%	−11.6%
	SLFBR	0.0300	0.0077	0.3117	−6.8%	−3.2%	−10.0%
	ILFL	0.0270	0.0103	0.3250	−5.8%	−3.5%	−9.4%
	CCF	0.0260	0.0112	0.2931	−6.8%	−4.4%	−8.8%
	SLFBL	0.0280	0.0117	0.2959	−7.0%	−8.1%	−7.9%
	UNCL	0.0270	0.0122	0.3178	−5.9%	−7.9%	−9.7%
Mean Diffusivity	CSTL	0.0440	0.0007	0.9503	11.2%	14.6%	6.9%
	CgLR	0.0350	0.0042	0.9471	7.4%	21.9%	15.8%
	ILFR	0.0300	0.0095	0.8716	9.4%	8.6%	6.7%

## 3. Results

### 3.1. Cohort characteristics

A total of 381 right-handed participants who underwent FBP, FTP, and brain MRI were included. There were 200 females and 181 males. The cohort was divided into four groups: FBP + /FTP+ (n = 38, F:M = 23:15), FBP + /FTP- (n = 133, F:M = 72:61), FBP-/FTP+ (n = 5, F:M = 1:4), and FBP-/FTP- (n = 205, F:M = 104:101). The mean age of each group (+/- standard deviation) was 73.9 ± 8.2, 77.1 ± 7.1, 77.2 ± 6.6, and 74.3 ± 7.5 years old, respectively (*p* < .01). There was no significant difference in the number of education years (FBP + /FTP + : 15.2 ± 2.5, FBP + /FTP-: 16.2 ± 2.4, FBP-/FTP + : 16.8 ± 2.7, FBP-/FTP-: 16.5 ± 2.6; *p* = .026). There was also no significant difference in the total intracranial volumes between the groups *(p = .*529). Significant differences were observed in the MMSE and MoCA scores between the groups *(p < .*001 for both) with the FBP + /FPT+ group having the lowest scores for both assessments (MMSE: 26.1 ± 3.5 and MoCA 19.0 ± 6.0), compared to the other groups (MMSE: 27.9 ± 2.2, 28.0 ± 1.4, 28.9 ± 1.4; MoCA: 23.8 ± 4.3, 25.2 ± 2.2, 25.3 ± 3.6, for FBP + /FTP-, FBP-/FTP + , and FBP-/FTP-, respectively)

### 3.2. Correlations between PET SUVR values and white matter microstructural changes

Significant negative correlations were observed between FBP SUVR and a subset of DTI metrics among the 381 participants. Specifically, CX, FN, and FA were significantly correlated with FBP SUVR (Table 1). FN showed the highest degree of correlations with beta coefficients ranging from −0.3991 to −0.2877 in the majority of white matter tracts examined (15 out of 18). The three tracts that showed no significant differences were the bilateral cingulum-hippocampus and right cingulum-cingulate gyrus tracts. While statistically significant, the beta-coefficients between FA and CX, and FBP SUVR were substantially smaller and close to 0, ranging from −0.0658 to −0.0269. Significant positive correlation was only observed between MD and FBP SUVR in the left corticospinal tract, with the beta-coefficient of 0.1105 (Table 1).

When females and males were examined separately, only females had tracts with positive correlations between MD and FBP SUVR (beta-coefficients 0.1208 to 0.1690) in the bilateral superior longitudinal fasciculi, corpus callosum-forceps minor, right inferior longitudinal fasciculus, and left corticospinal tract (S2 Table). Interestingly, none of the tracts showed significant correlations between MD and FBP SUVR in males. Instead, males showed significant negative correlations in FN (beta-coefficients −0.3977 to −0.3867) in the left inferior fronto-occipital fasciculus, left anterior thalamic radiations, and left inferior longitudinal fasciculus.

In general, a lower degree of correlation was observed between FTP SUVR and DTI metrics. For the entire cohort, no significant correlations were found between FTP SUVR and DTI metrics. For females only, significant positive correlations were observed between MD and FTP SUVR in two tracts, right inferior longitudinal fasciculus and left superior longitudinal fasciculus, with the beta-coefficients of 0.2643 and 0.1610, respectively (*p* < .05). In males only, AFD showed significant negative correlation in the left corticospinal tract with the beta-coefficient of 0.1040 (*p* < .05).

### 3.3. PET positivity and white matter microstructural changes

**Fig 1:**
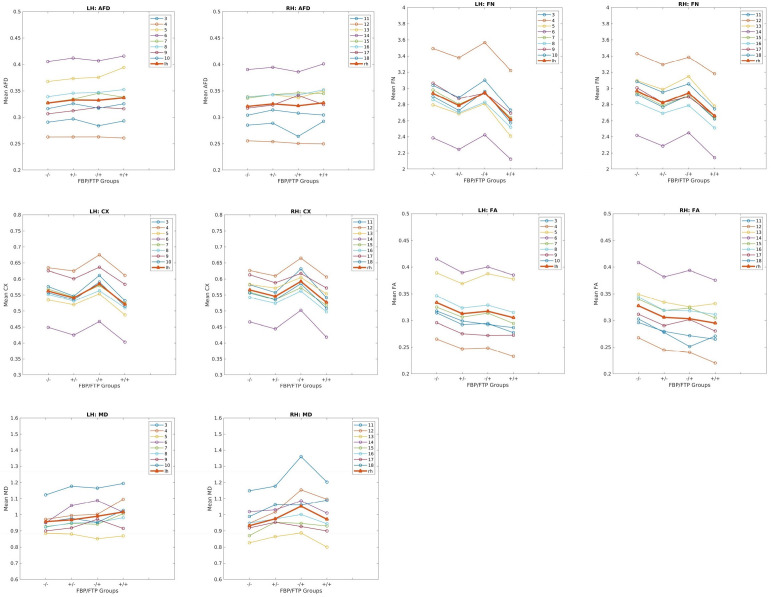
Mean DTI metrics in the left vs right hemisphere for each PET group. AFD = apparent fiber density, CX = complexity, MD = mean diffusivity, FA = fractional anisotropy, FN = fixel number, lh = mean values of the left hemisphere; rh = mean values of the right hemisphere, LH = left hemisphere; RH = right hemisphere. The tract names corresponding to the numbers are listed in the S1 Table.

To determine whether single or double positivity in PET is more strongly correlated with the microscopic white matter changes, the cohort was divided into four groups based on PET positivity as described previously. Between-group comparisons of each DTI metric among the four groups revealed that double PET positivity (i.e., FBP + /FTP+) is generally associated with the greatest reductions in CX, FN, and FA, compared to single PET-positive or PET-negative groups (Fig 1, Table 2). Table 2 demonstrates the percent changes in the value of DTI metrics for individual tracts compared to PET-negative cohort that are statistically significant (Bonferroni-adjusted *p* < .0125). For instance, the greatest reduction in DTI metrics was seen with FA of the right cingulum-hippocampus tract, where FBP + /FTP+ showed a 17.7% decrease compared to FBP-/FTP-. Single PET-positive groups, FBP + /FTP- and FBP-/FTP + , showed 8.6% and 10.2% reductions, respectively. Interestingly, there were exceptions to the general trend of reduced DTI metrics in PET-positive groups. CX of the left anterior thalamic radiations, corpus callosum-forceps minor, and right corticospinal tract as well as FN of the left anterior thalamic radiations showed an increase in respective DTI metrics in FTP-positive-only (FBP-/FTP+) group, compared to the PET-negative group. However, the size of the FBP-/FTP+ group is too small (N = 5) to draw any conclusions about the relationship between CX and FTP positivity. MD was increased in PET-positive groups in a subset of tracts, including the left corticospinal tract, right cingulum-hippocampus, and right inferior longitudinal fasciculus. However, there was no conspicuous trend in MD changes between single PET-positive and double PET-positive groups.

Sex differences in DTI metrics and PET positivity were also observed. Only females showed a significant increase in MD of various fiber tracts (bilateral superior longitudinal fasciculi, left anterior thalamic radiations, right inferior longitudinal fasciculus, corpus callosum-forceps minor, and left corticospinal tract) in PET-positive groups compared to the PET-negative group (S3 Table). Only males showed signification reduction in FA in two tracts, right cingulum-hippocampus and right corticospinal tract (S3 Table).

### 3.4. Associations between white matter microstructural changes and cognitive impairment

We next asked whether there are any associations between the microstructural white matter changes and AD symptoms, specifically cognitive impairment. Two different assessments of global cognitive impairment were used—MMSE and MoCA—as prior studies have shown that they have variable degrees of sensitivity and accuracy for the detection of AD symptoms [33–35]. Cognitive impairment was defined as MMSE ≤ 26 [36] and MoCA < 26 [37]. In general, MoCA scores were correlated with a greater number of significant changes in DTI metrics in various tracts (Table 3). Particularly, FA in nearly all of the tracts (17 out of 18) showed significant positive correlations with MoCA < 26. Significant negative correlations were observed between MD and both MMSE ≤ 26 and MoCA < 26, but the degree of correlation was higher for MoCA (beta-coefficients of −2.0742 to −2.5974) than for MMSE (−0.9423 to −0.3357). Comparisons between the sexes showed that females have a substantially greater number fiber tracts demonstrating significant positive correlations between FA and cognitive impairment as measured by MoCA and MMSE as well as significant negative correlations between MD and cognitive impairment (S4 and S5 Tables).

**Table 3:** Correlation between DTI metrics and cognitive impairment (*p* < .05 only).

MMSE ≤ 26							
**Metric**	**Fiber Tract**	**ß-Coefficient**	**CI low**	**CI high**	***p-*value**	**R2**	**Adjusted R2**
Complexity	CCO	1.7117	0.2082	3.2152	0.0258	0.5332	0.5179
Mean Diffusivity	UNCR	−0.6020	−1.0100	−0.1940	0.0039	0.5453	0.5298
	IFOL	−0.5730	−1.0129	−0.1331	0.0108	0.5434	0.5276
	ILFR	−0.9423	−1.6687	−0.2159	0.0112	0.5385	0.5228
	CSTL	−0.8176	−1.4885	−0.1467	0.0171	0.5376	0.5216
	IFOR	−0.4096	−0.7743	−0.0450	0.0278	0.5385	0.5227
	CgLR	−0.6030	−1.1757	−0.0303	0.0391	0.5292	0.5130
	CgLL	−0.3357	−0.6646	−0.0068	0.0455	0.5367	0.5208
	ILFL	−0.6106	−1.2102	−0.0110	0.0460	0.5352	0.5192
**MoCA < 26**							
**Metric**	**Fiber Tract**	**ß-Coefficient**	**CI low**	**CI high**	***p-*value**	**R2**	**Adjusted R2**
Apparent Fiber Density	CgUL	−4.4851	−8.7507	−0.2194	0.0394	0.4408	0.4225
	CSTL	−4.3001	−8.5593	−0.0409	0.0479	0.4403	0.4220
	CgUR	−4.2696	−8.5360	−0.0033	0.0498	0.4402	0.4219
Fractional Anisotropy	IFOL	10.3239	5.0892	15.5587	0.0001	0.4566	0.4388
	ILFR	10.1404	4.8877	15.3931	0.0002	0.4557	0.4379
	ILFL	10.3398	4.9442	15.7355	0.0002	0.4554	0.4376
	UNCL	9.8141	4.5724	15.0558	0.0003	0.4545	0.4366
	ATRL	9.2462	4.2142	14.2781	0.0003	0.4538	0.4359
	CCO	8.4255	3.7505	13.1005	0.0004	0.4531	0.4351
	IFOR	9.3682	4.1044	14.6320	0.0005	0.4526	0.4347
	CgLR	8.6662	3.7961	13.5364	0.0005	0.4526	0.4347
	CSTR	8.3055	3.0334	13.5776	0.0021	0.4488	0.4307
	ATRR	8.4636	3.0276	13.8996	0.0024	0.4484	0.4304
	SLFBR	7.8758	2.7050	13.0465	0.0029	0.4478	0.4297
	CCF	7.8606	2.2648	13.4563	0.0060	0.4459	0.4277
	CgLL	7.1486	2.0461	12.2511	0.0062	0.4458	0.4276
	SLFBL	7.6993	2.1360	13.2626	0.0068	0.4455	0.4274
	CSTL	6.8892	1.7275	12.0508	0.0090	0.4448	0.4266
	UNCR	6.4825	1.3017	11.6633	0.0143	0.4435	0.4253
	CgUL	4.1177	0.0680	8.1675	0.0463	0.4404	0.4221
Mean Diffusivity	CgLR	−2.5974	−3.9076	−1.2872	0.0001	0.4525	0.4337
	ILFL	−2.0742	−3.4605	−0.6878	0.0035	0.4430	0.4238
	ILFR	−2.3606	−4.0448	−0.6764	0.0061	0.4423	0.4233

## 4. Discussion

### 4.1. Direct correlations between DTI metrics and levels of FBP and FTP

Direct comparisons between the DTI metrics and levels of FBP and FTP SUVR revealed substantially greater correlations with FBP than FTP. Both voxel-based metrics, FA and MD, and fixel-based metrics, CX and FN, showed statistically significant correlations with FBP SUVR (Table 1). Among the DTI metrics, FN showed the greatest degree of negative correlations with the FBP levels, suggesting that elevated amyloid-beta is correlated with the reduction in the number of white matter fiber bundles. A positive correlation between MD and FBP SUVR was observed in a single tract, the left corticospinal tract, additionally reflecting an increased damage to the white matter tracts in association with elevated amyloid-beta. In comparison, no DTI metrics were significantly correlated with FTP SUVR for the whole cohort.

Previous studies examining the correlations between amyloid-beta and white matter microstructures have shown conflicting results. Several studies demonstrated no significant correlations between white matter microstructures, as assessed by DTI- or other fixel-based analysis, and levels of amyloid-beta [17,19,38]. Strain *et al.*, for instance, saw associations between tau proteins and anterior temporal white matter integrity, but not amyloid-beta [17]. Other studies have shown significant correlations, including a study by Collij *et al.*, which found decreased FA and increased MD as well as radial diffusion and axial diffusivity associated with high amyloid burden [18]. These differences may be attributed to the differences in cohort, scanner types, image acquisition parameters, and analysis methods. It should also be noted that the beta-coefficients for CX and FA, while statistically significant, were close to 0, suggestive of weak to negligible correlation.

### 4.2. DTI metrics changes associated with single vs double PET positivity

Group-wise comparisons between DTI metrics and PET positivity revealed a widespread reduction in FA across 14 out of 18 tracts examined in PET-positive groups, compared to the PET-negative group (Table 2). With the exception of the right anterior thalamic radiations, FA was most reduced in the double PET-positive group (FBP + /FTP+), compared to the single PET-positive groups (FBP + /FBP- and FBP-/FTP+), in keeping with the hypothesis that double PET-positive individuals have a more profound disruption in the white matter microstructure. Fewer tracts were associated with a decrease in CX and FN in association with PET-positivity, but the FBP + /FTP+ group still showed the greatest degree of reduction compared to the other groups. These results support the previous findings that demonstrated increased brain atrophy and increased risk of dementia in individuals with elevations in both amyloid-beta and tau [27, 39,40].

In our cohort, there were only 5 subjects who were FBP-/FTP + . This is not unexpected as individuals who are amyloid-beta negative and tau-positive are shown to represent only 4.1% of the cognitive unimpaired population [41]. Subsequently, relatively little is known about the associated structural abnormalities, including the white matter microstructural changes. Our results showed generally less reduction in FA, FX, and CX in FBP-/FTP+ compared to FBP + /FTP- or FBP + /FTP + . Unexpectedly, CX and FN in a subset of tracts were increased in FBP-FTP+ compared to FBP-/FTP- (Table 2). The size of the FBP-/FTP+ group is too small to draw any conclusions about whether these observations are truly significant and reflect an underlying compensatory mechanism. More studies, such a meta-analysis of pooled data, are needed for further assessment of the brain changes in FBP-/FTP+ individuals.

### 4.3. Correlations with cognitive impairment

We also explored the potential role of DTI metrics as imaging biomarkers that may supplement the assessment of cognitive impairment. To this end, we examined associations between DTI metrics and MMSE and MoCA, both of which are widely used screening tools for cognitive impairment. Two separate assessment tools were examined as previous studies have shown different sensitivities to cognitive impairment [33,35]. MoCA is thought to have superior accuracy for detecting cognitive impairment [33,35], but MMSE remains one of the best known and commonly used screening tools for cognitive impairment clinically [42]. We sought to investigate whether there are also differences in the degree of correlation with white matter disruption between the two assessments.

In our study, cognitive impairment as noted by the MoCA score < 26 was associated with greater correlations with DTI metrics, compared to MMSE ≤ 26. Positive correlations between FA and MoCA were observed in nearly all of the tracts, while no tracts showed significant correlations between FA and MMSE (Table 3). The findings are also in line with previous studies that showed FA to be a strong predictor of cognitive impairment. For instance, Xing *et al*. showed that the mean FA value was significantly associated with the MMSE scores [14]. Mayo *et al.* found a significant positive correlation between FA and memory scores in both AD and healthy older adults [15]. Negative correlations between MD and MoCA were seen in a subset of tracts, but the degree of correlations were higher for MoCA than MMSE, as discussed in the results section. Overall, these findings support the previous findings that MoCA is more sensitive and accurate in discerning early cognitive impairment [33,35]. It should be noted that our results are limited to the correlations with the global assessment of cognitive impairment. Further studies are needed to determine whether there are specific correlation between individual DTI metrics and different cognitive domains.

### 4.4. Sex differences

Several notable differences in DTI changes were observed between females and males. One of the most skewed DTI metrics between the sexes was MD, where only females showed positive correlations with FBP/FTP SUVR levels and PET-positivity. In particular, the right inferior longitudinal fasciculus and the left superior longitudinal fasciculus showed consistent positive correlations with FBP SUVR, FTP SUVR, and PET-positivity. In comparison, only males showed statistically significant, negative correlations between FN and FBP SUVR and negative correlations between FA and PET-positivity (S2 and S3 Tables). A prior study using free-water-corrected DTI showed lower FA and higher radial diffusivity in female AD patients [22], but our understanding of the sex differences in DTI metrics in AD remains overall limited. Our results demonstrate additional types of white matter disruption that are differentially affected in females and males in association with elevated amyloid-beta and tau.

Sex differences were also observed when the correlations between DTI metrics and cognitive impairment were examined. In general, females showed greater correlations between DTI metrics, particularly FA and MD, and both MoCA and MMSE (S4 and S5 Tables). Only males showed significant, negative correlations between AFD in the bilateral cingulum-cingulate gyrus tracts for both MoCA and MMSE. Clinically, females with mild cognitive impairment exhibit more pronounced cognitive decline [43], and studies have shown that females progress faster to the clinical diagnosis of mild cognitive impairment and dementia [44]. Our results suggest that more widespread white matter microstructural changes in females may be potentially connected to the sex differences in cognitive impairment in AD. Sex differences in DTI changes and specific cognitive domains require further studies, however.

### 4.5. Limitations

The current study has several limitations. This is a retrospective study, which only included baseline imaging studies and cognitive assessments. Therefore, it is difficult to make longitudinal assessments and draw any conclusions on how the observed white matter microstructure changes affect AD progression. Furthermore, it is uncertain if and to what extent the white matter changes precede clinical symptoms. PET groups were not evenly distributed in terms of participant numbers, particularly for the FBP-/FTP+ group that had only 5 participants. Some of the clinical data such as co-morbidities and genetic data were not included in the study. While studies have shown strong correlations between FTP and underlying tau pathology in AD, which is characterized by pair helical filament consisting of three-repeat tau and four-repeat tau, there are limitations to the specificity of FTP [45]. For instance, a histopathological study showed that regions with FTP uptake and histopathological tau burden may not match in the presence of secondary tau pathology [46]. Furthermore, there is evidence that FTP may bind to three-repeat tau and four-repeat tau species in frontotemporal lobar degeneration, suggesting that there is overlap in FTP binding of tau associated with AD and other tau species [45].

Lastly, this study only used DTI-based metrics for the assessment of white matter changes. There are other modeling techniques derived from diffusion MRI, such as the composite hindered and restricted model of diffusion (CHARMED) and neurite orientation dispersion and density imaging (NODDI; list of acronyms in S6 Table), that allow characterization of subtle microstructural changes and crossing white matter fibers [47,48]. DTI is generally thought to be more limited in the characterization of complex white matter structures with crossing fibers, although newer, fixel-based analysis allows for more detailed characterization of individual fiber population within a voxel [47]. A more comprehensive study employing various diffusion MRI modeling techniques may help not only understand the strengths and limitations of individual techniques but also allow more accurate assessment of microstructural white matter changes associated with AD.

## 5. Conclusions

Distinctive associations between FBP/FTP levels and voxel-based analysis and fixel-based analysis derived DTI metrics were observed. FN exhibited the greatest degree of negative correlations with FBP SUVR, but not with FTP SUVR. In general, double positivity in FBP and FTP showed the greatest changes in DTI metrics, suggestive of progressive microstructural white matter disruption across the AD continuum. Widespread changes in FA were positively correlated with cognitive impairment, with stronger associations seen with MoCA scores than MMSE. Substantial sex differences were found in the degree and type of white matter disruption associated with elevated FBP/FTP and cognitive impairment. Taken together, these findings support the potential role of white matter microstructure changes as early AD biomarkers, highlight underlying sex differences, and underscore the need for further studies.

## Supporting information

S1 Table White matter tracts and abbreviations. (DOCX)

S2 Table Correlations between FBP SUVR and DTI (*p* < .05 only): Female vs Male. (DOCX)

S3 Table Group-wise comparisons between DTI metrics and PET positivity (*p* < .0125 only): Female vs Male. (DOCX)

S4 Table Correlation between DTI metrics and MMSE: Female vs Male. (DOCX)

S5 Table Correlation between DTI metrics and MoCA: Female vs Male (*p* < .05 only). (DOCX)

S6 Table List of acronyms. (DOCX)
