# Supplementary material for: White matter microstructure disruption associated with PET and cognitive impairment in Alzheimer’s disease
Source: PLoS One. 2026 Apr 8;21(4):e0346661. doi: 10.1371/journal.pone.0346661 (PMC13061220; doi:10.1371/journal.pone.0346661)
Supplement: S1 Table — (DOCX) [file pone.0346661.s001.docx]

**Table S1. White matter tracts and abbreviations**

|  | Abbreviations | White matter tracts |
| --- | --- | --- |
| 1 | CCO | Corpus Callosum - Forceps Major |
| 2 | CCF | Corpus Callosum - Forceps Minor |
| 3 | ATRL | Left Anterior Thalamic Radiations |
| 4 | CgLL | Left Cingulum - Hippocampus |
| 5 | CgUL | Left Cingulum - Cingulate Gyrus |
| 6 | CSTL | Left Corticospinal Tract |
| 7 | ILFL | Left Inferior Longitudinal Fasciculus |
| 8 | IFOL | Left Inferior Fronto-occipital Fasciculus |
| 9 | SLFBL | Left Superior Longitudinal Fasciculus |
| 10 | UNCL | Left Uncinate Fasciculus |
| 11 | ATRR | Right Anterior Thalamic Radiations |
| 12 | CgLR | Right Cingulum - Hippocampus |
| 13 | CgUR | Right Cingulum - Cingulate Gyrus |
| 14 | CSTR | Right Corticospinal Tract |
| 15 | ILFR | Right Inferior Longitudinal Fasciculus |
| 16 | IFOR | Right Inferior Fronto-occipital Fasciculus |
| 17 | SLFBR | Right Superior Longitudinal Fasciculus |
| 18 | UNCR | Right Uncinate Fasciculus |
