# Supplementary material for: White matter microstructure disruption associated with PET and cognitive impairment in Alzheimer’s disease
Source: PLoS One. 2026 Apr 8;21(4):e0346661. doi: 10.1371/journal.pone.0346661 (PMC13061220; doi:10.1371/journal.pone.0346661)
Supplement: S2 Table — (DOCX) [file pone.0346661.s002.docx]

**Table S2. Correlations between FBP SUVR and DTI (*p* < .05 only): Female vs Male**

| Female | | | | |
| --- | --- | --- | --- | --- |
| **Metric** | **Fiber Tract** | **β-Coefficient** | **R2** | ***p-*value** |
| Complexity | CSTL | -0.0612 | 0.1046 | 0.0368 |
| Fractional  Anisotropy | ATRR | -0.0583 | 0.1399 | 0.0022 |
|  | CSTR | -0.0603 | 0.1356 | 0.0032 |
|  | CCF | -0.0484 | 0.1592 | 0.0096 |
|  | IFOR | -0.0526 | 0.1592 | 0.0120 |
|  | IFOL | -0.0455 | 0.1700 | 0.0224 |
|  | CSTL | -0.0449 | 0.1363 | 0.0297 |
| Mean Diffusivity | SLFBL | 0.1473 | 0.1528 | 0.0013 |
|  | SLFBR | 0.1208 | 0.1572 | 0.0130 |
|  | CCF | 0.1488 | 0.2395 | 0.0149 |
|  | ILFR | 0.1690 | 0.1467 | 0.0208 |
|  | CSTL | 0.1370 | 0.2152 | 0.0386 |
| **Male** | | | | |
| **Metric** | **Fiber Tract** | **β-Coefficient** | **R2** | ***p-*value** |
| Complexity | CCF | -0.0723 | 0.0960 | 0.0188 |
|  | ATRL | -0.0714 | 0.0896 | 0.0206 |
|  | IFOL | -0.0606 | 0.0991 | 0.0249 |
|  | IFOR | -0.0622 | 0.0877 | 0.0291 |
|  | ILFR | -0.0606 | 0.0793 | 0.0349 |
|  | UNCL | -0.0632 | 0.1164 | 0.0358 |
|  | ATRR | -0.0611 | 0.0958 | 0.0496 |
| Fixel Number | IFOL | -0.3977 | 0.0781 | 0.0292 |
|  | ATRL | -0.3782 | 0.0652 | 0.0398 |
|  | ILFL | -0.3867 | 0.0682 | 0.0424 |
| Fractional  Anisotropy | CgLR | -0.0438 | 0.1921 | 0.0334 |
