# Supplementary material for: White matter microstructure disruption associated with PET and cognitive impairment in Alzheimer’s disease
Source: PLoS One. 2026 Apr 8;21(4):e0346661. doi: 10.1371/journal.pone.0346661 (PMC13061220; doi:10.1371/journal.pone.0346661)
Supplement: S3 Table — (DOCX) [file pone.0346661.s003.docx]

**Table S3. Group-wise comparisons between DTI metrics and PET positivity (*p* < .0125 only): Female vs Male.**

| **Female** | | | | | | | |
| --- | --- | --- | --- | --- | --- | --- | --- |
| **Metric** | **Fiber Tract** | **R2** | ***p-*value** | **FBP- FTP-  (n = 101)** | **FBP+ FTP-  (n = 61)** | **FBP- FTP+  (n = 4)** | **FBP+ FTP+  (n = 15)** |
| Mean  Diffusivity | CCF | 0.0910 | 0.0007 | 1.0566 | 12.5% | 2.0% | 10.2% |
|  | ATRL | 0.0850 | 0.0014 | 1.1337 | 16.2% | 1.4% | 6.5% |
|  | SLFBR | 0.0800 | 0.0026 | 0.8636 | 11.5% | 5.2% | 4.5% |
|  | CSTL | 0.0750 | 0.0028 | 0.9618 | 12.2% | 14.4% | 14.5% |
|  | ILFR | 0.0640 | 0.0100 | 0.8751 | 15.3% | 7.7% | 10.0% |
|  | SLFBL | 0.0650 | 0.0110 | 0.8688 | 9.4% | 7.3% | 6.5% |
| **Male** | | | | | | | |
| **Metric** | **Fiber Tract** | **R2** | ***p-*value** | **FBP- FTP-  (n = 105)** | **FBP+ FTP-  (n = 71)** | **FBP- FTP+  (n = 1)** | **FBP+ FTP+  (n = 23)** |
| Fractional  Anisotropy | CgLR | 0.0680 | 0.0014 | 0.2713 | -10.0% | -0.5% | -20.6% |
|  | CSTR | 0.0520 | 0.0100 | 0.4124 | -7.5% | -8.6% | -6.4% |
