# Supplementary material for: White matter microstructure disruption associated with PET and cognitive impairment in Alzheimer’s disease
Source: PLoS One. 2026 Apr 8;21(4):e0346661. doi: 10.1371/journal.pone.0346661 (PMC13061220; doi:10.1371/journal.pone.0346661)
Supplement: S4 Table — (DOCX) [file pone.0346661.s004.docx]

**Table S4. Correlation between DTI metrics and MMSE: Females vs Males (*p* < .05 only)**

| **Female** | | | | | | | |
| --- | --- | --- | --- | --- | --- | --- | --- |
| **Metric** | **Fiber Tract** | **ß-Coefficient** | **CI low** | **CI high** | ***p-*value** | **R2** | **Adjusted R2** |
| Fractional Anisotropy | CCO | 6.8301 | 3.4212 | 10.2389 | 0.0001 | 0.1487 | 0.1194 |
|  | IFOR | 7.6824 | 3.6587 | 11.7061 | 0.0002 | 0.1422 | 0.1126 |
|  | CSTL | 7.5530 | 3.5057 | 11.6003 | 0.0003 | 0.1393 | 0.1096 |
|  | IFOL | 7.5640 | 3.3570 | 11.7710 | 0.0005 | 0.1348 | 0.1050 |
|  | CSTR | 7.3560 | 3.2169 | 11.4951 | 0.0006 | 0.1335 | 0.1036 |
|  | ILFR | 7.4528 | 3.2389 | 11.6667 | 0.0006 | 0.1329 | 0.1030 |
|  | ILFL | 7.3882 | 2.9539 | 11.8226 | 0.0012 | 0.1265 | 0.0964 |
|  | ATRL | 5.8245 | 1.5458 | 10.1031 | 0.0079 | 0.1092 | 0.0785 |
|  | UNCL | 5.6612 | 1.2905 | 10.0319 | 0.0114 | 0.1058 | 0.0750 |
|  | ATRR | 5.6421 | 1.0006 | 10.2836 | 0.0175 | 0.1019 | 0.0710 |
|  | SLFBR | 4.8544 | 0.8182 | 8.8906 | 0.0187 | 0.1013 | 0.0703 |
|  | UNCR | 5.1162 | 0.7592 | 9.4733 | 0.0216 | 0.1000 | 0.0690 |
|  | CgUL | 3.6184 | 0.5291 | 6.7077 | 0.0220 | 0.0999 | 0.0688 |
|  | CCF | 5.1132 | 0.4826 | 9.7438 | 0.0306 | 0.0969 | 0.0657 |
|  | CgLR | 4.0622 | 0.0493 | 8.0751 | 0.0473 | 0.0930 | 0.0618 |
| Mean Diffusivity | CSTL | -3.1510 | -4.4902 | -1.8118 | 0.0000 | 0.1913 | 0.1610 |
|  | ATRL | -1.9466 | -3.1038 | -0.7895 | 0.0011 | 0.1443 | 0.1112 |
|  | IFOL | -1.3757 | -2.2048 | -0.5466 | 0.0013 | 0.1475 | 0.1159 |
|  | ILFR | -1.9808 | -3.2548 | -0.7069 | 0.0025 | 0.1272 | 0.0951 |
|  | ILFL | -1.5739 | -2.6069 | -0.5408 | 0.0030 | 0.1401 | 0.1085 |
|  | CCO | -1.6617 | -2.7611 | -0.5623 | 0.0033 | 0.1334 | 0.1000 |
|  | CgLR | -1.3601 | -2.3620 | -0.3581 | 0.0081 | 0.1289 | 0.0969 |
|  | SLFBL | -2.6726 | -4.7300 | -0.6153 | 0.0112 | 0.1081 | 0.0746 |
|  | UNCR | -1.2019 | -2.1372 | -0.2667 | 0.0121 | 0.1156 | 0.0833 |
|  | CCF | -2.0016 | -3.5853 | -0.4180 | 0.0136 | 0.1205 | 0.0865 |
|  | CgUL | -1.6043 | -2.8906 | -0.3181 | 0.0148 | 0.1247 | 0.0923 |
|  | SLFBR | -2.4577 | -4.4369 | -0.4785 | 0.0153 | 0.1129 | 0.0798 |
|  | CgLL | -0.5210 | -1.0180 | -0.0241 | 0.0400 | 0.1194 | 0.0870 |
| **Male** | | | | | | | |
| **Metric** | **Fiber Tract** | **ß-Coefficient** | **CI low** | **CI high** | ***p-*value** | **R2** | **Adjusted R2** |
| Apparent Fiber Density | CgUR | -4.6969 | -8.8477 | -0.5461 | 0.0268 | 0.1130 | 0.0850 |
|  | CgUL | -4.0951 | -8.1103 | -0.0799 | 0.0457 | 0.1080 | 0.0800 |
| Complexity | CgUL | 3.1759 | 0.1161 | 6.2357 | 0.0420 | 0.1090 | 0.0810 |
| Mean Diffusivity | UNCL | -2.1797 | -3.9604 | -0.3989 | 0.0167 | 0.1160 | 0.0880 |
