# Supplementary material for: White matter microstructure disruption associated with PET and cognitive impairment in Alzheimer’s disease
Source: PLoS One. 2026 Apr 8;21(4):e0346661. doi: 10.1371/journal.pone.0346661 (PMC13061220; doi:10.1371/journal.pone.0346661)
Supplement: S5 Table — (DOCX) [file pone.0346661.s005.docx]

**Table S5. Correlation between DTI metrics and MoCA: Female vs Male (*p* < 0.05 only)**

| **Female** | | | | | | | |
| --- | --- | --- | --- | --- | --- | --- | --- |
| **Metric** | **Fiber Tract** | **ß-Coefficient** | **CI low** | **CI high** | ***p-*value** | **R2** | **Adjusted R2** |
| Fractional Anisotropy | CSTR | 19.4315 | 11.1642 | 27.6988 | 0.0000 | 0.1942 | 0.1662 |
|  | CSTL | 18.6856 | 10.5536 | 26.8176 | 0.0000 | 0.1902 | 0.1621 |
|  | ILFR | 19.1170 | 10.6677 | 27.5663 | 0.0000 | 0.1876 | 0.1594 |
|  | IFOR | 17.1921 | 9.0238 | 25.3605 | 0.0001 | 0.1761 | 0.1475 |
|  | CCO | 14.5101 | 7.5566 | 21.4635 | 0.0001 | 0.1748 | 0.1462 |
|  | ATRL | 17.3394 | 8.7886 | 25.8901 | 0.0001 | 0.1707 | 0.1420 |
|  | ILFL | 17.5079 | 8.5208 | 26.4949 | 0.0002 | 0.1653 | 0.1363 |
|  | UNCL | 16.7396 | 7.9789 | 25.5004 | 0.0002 | 0.1628 | 0.1337 |
|  | IFOL | 16.3506 | 7.7769 | 24.9243 | 0.0002 | 0.1625 | 0.1335 |
|  | CgLR | 15.2002 | 7.2062 | 23.1942 | 0.0002 | 0.1621 | 0.1331 |
|  | ATRR | 15.4591 | 6.0729 | 24.8454 | 0.0014 | 0.1461 | 0.1165 |
|  | CCF | 14.0666 | 4.6933 | 23.4399 | 0.0035 | 0.1377 | 0.1077 |
|  | UNCR | 13.1237 | 4.2844 | 21.9631 | 0.0038 | 0.1368 | 0.1068 |
|  | SLFBR | 11.9282 | 3.7273 | 20.1292 | 0.0046 | 0.1351 | 0.1051 |
|  | CgLL | 11.7465 | 3.3232 | 20.1698 | 0.0065 | 0.1319 | 0.1018 |
|  | SLFBL | 9.9252 | 0.7638 | 19.0866 | 0.0339 | 0.1173 | 0.0866 |
| Mean Diffusivity | ILFL | -4.9278 | -7.0429 | -2.8127 | 0.0000 | 0.2206 | 0.1918 |
|  | CgLR | -4.3470 | -6.4135 | -2.2805 | 0.0001 | 0.1997 | 0.1701 |
|  | IFOL | -2.8680 | -4.6120 | -1.1241 | 0.0014 | 0.1799 | 0.1493 |
|  | CgUL | -4.3005 | -6.9933 | -1.6077 | 0.0019 | 0.1705 | 0.1396 |
|  | ILFR | -3.9119 | -6.5645 | -1.2593 | 0.0041 | 0.1455 | 0.1138 |
|  | ATRL | -3.2757 | -5.7172 | -0.8342 | 0.0089 | 0.1558 | 0.1229 |
|  | CSTL | -3.7404 | -6.6471 | -0.8337 | 0.0120 | 0.1523 | 0.1203 |
|  | CCF | -4.1781 | -7.4815 | -0.8747 | 0.0135 | 0.1560 | 0.1231 |
|  | IFOR | -1.2693 | -2.4034 | -0.1352 | 0.0285 | 0.1539 | 0.1223 |
|  | CCO | -2.3854 | -4.7104 | -0.0605 | 0.0444 | 0.1420 | 0.1088 |
| **Male** | | | | | | | |
| **Metric** | **Fiber Tract** | **ß-Coefficient** | **CI low** | **CI high** | ***p-*value** | **R2** | **Adjusted R2** |
| Apparent Fiber Density | CgUL | -9.8266 | -17.8081 | -1.8452 | 0.0161 | 0.2050 | 0.1802 |
|  | CgUR | -10.0063 | -18.2827 | -1.7299 | 0.0181 | 0.2042 | 0.1793 |
|  | CSTR | -8.6493 | -16.1533 | -1.1453 | 0.0241 | 0.2021 | 0.1771 |
|  | CSTL | -8.2596 | -15.6698 | -0.8494 | 0.0291 | 0.2007 | 0.1757 |
| Fractional Anisotropy | CCO | 11.8559 | 3.2605 | 20.4513 | 0.0071 | 0.2110 | 0.1864 |
|  | IFOL | 10.8936 | 1.8547 | 19.9325 | 0.0184 | 0.2040 | 0.1791 |
|  | ATRL | 9.8037 | 1.4659 | 18.1414 | 0.0214 | 0.2029 | 0.1780 |
|  | ILFL | 10.1585 | 0.9393 | 19.3778 | 0.0310 | 0.2003 | 0.1753 |
| Mean Diffusivity | CgLR | -3.3776 | -5.7197 | -1.0356 | 0.0049 | 0.2050 | 0.1793 |
|  | CgLL | -2.0369 | -3.9156 | -0.1582 | 0.0337 | 0.1979 | 0.1722 |
