# Supplementary material for: White matter microstructure disruption associated with PET and cognitive impairment in Alzheimer’s disease
Source: PLoS One. 2026 Apr 8;21(4):e0346661. doi: 10.1371/journal.pone.0346661 (PMC13061220; doi:10.1371/journal.pone.0346661)
Supplement: S6 Table — (DOCX) [file pone.0346661.s006.docx]

**Table S1. White matter tracts and abbreviations**

|  | Abbreviations | White matter tracts |
| --- | --- | --- |
| 1 | CCO | Corpus Callosum - Forceps Major |
| 2 | CCF | Corpus Callosum - Forceps Minor |
| 3 | ATRL | Left Anterior Thalamic Radiations |
| 4 | CgLL | Left Cingulum - Hippocampus |
| 5 | CgUL | Left Cingulum - Cingulate Gyrus |
| 6 | CSTL | Left Corticospinal Tract |
| 7 | ILFL | Left Inferior Longitudinal Fasciculus |
| 8 | IFOL | Left Inferior Fronto-occipital Fasciculus |
| 9 | SLFBL | Left Superior Longitudinal Fasciculus |
| 10 | UNCL | Left Uncinate Fasciculus |
| 11 | ATRR | Right Anterior Thalamic Radiations |
| 12 | CgLR | Right Cingulum - Hippocampus |
| 13 | CgUR | Right Cingulum - Cingulate Gyrus |
| 14 | CSTR | Right Corticospinal Tract |
| 15 | ILFR | Right Inferior Longitudinal Fasciculus |
| 16 | IFOR | Right Inferior Fronto-occipital Fasciculus |
| 17 | SLFBR | Right Superior Longitudinal Fasciculus |
| 18 | UNCR | Right Uncinate Fasciculus |

**Table S2. Correlations between FBP SUVR and DTI (*p* < .05 only): Female vs Male**

| Female | | | | |
| --- | --- | --- | --- | --- |
| **Metric** | **Fiber Tract** | **β-Coefficient** | **R2** | ***p-*value** |
| Complexity | CSTL | -0.0612 | 0.1046 | 0.0368 |
| Fractional  Anisotropy | ATRR | -0.0583 | 0.1399 | 0.0022 |
|  | CSTR | -0.0603 | 0.1356 | 0.0032 |
|  | CCF | -0.0484 | 0.1592 | 0.0096 |
|  | IFOR | -0.0526 | 0.1592 | 0.0120 |
|  | IFOL | -0.0455 | 0.1700 | 0.0224 |
|  | CSTL | -0.0449 | 0.1363 | 0.0297 |
| Mean Diffusivity | SLFBL | 0.1473 | 0.1528 | 0.0013 |
|  | SLFBR | 0.1208 | 0.1572 | 0.0130 |
|  | CCF | 0.1488 | 0.2395 | 0.0149 |
|  | ILFR | 0.1690 | 0.1467 | 0.0208 |
|  | CSTL | 0.1370 | 0.2152 | 0.0386 |
| **Male** | | | | |
| **Metric** | **Fiber Tract** | **β-Coefficient** | **R2** | ***p-*value** |
| Complexity | CCF | -0.0723 | 0.0960 | 0.0188 |
|  | ATRL | -0.0714 | 0.0896 | 0.0206 |
|  | IFOL | -0.0606 | 0.0991 | 0.0249 |
|  | IFOR | -0.0622 | 0.0877 | 0.0291 |
|  | ILFR | -0.0606 | 0.0793 | 0.0349 |
|  | UNCL | -0.0632 | 0.1164 | 0.0358 |
|  | ATRR | -0.0611 | 0.0958 | 0.0496 |
| Fixel Number | IFOL | -0.3977 | 0.0781 | 0.0292 |
|  | ATRL | -0.3782 | 0.0652 | 0.0398 |
|  | ILFL | -0.3867 | 0.0682 | 0.0424 |
| Fractional  Anisotropy | CgLR | -0.0438 | 0.1921 | 0.0334 |

**Table S3. Group-wise comparisons between DTI metrics and PET positivity (*p* < .0125 only): Female vs Male.**

| **Female** | | | | | | | |
| --- | --- | --- | --- | --- | --- | --- | --- |
| **Metric** | **Fiber Tract** | **R2** | ***p-*value** | **FBP- FTP-  (n = 101)** | **FBP+ FTP-  (n = 61)** | **FBP- FTP+  (n = 4)** | **FBP+ FTP+  (n = 15)** |
| Mean  Diffusivity | CCF | 0.0910 | 0.0007 | 1.0566 | 12.5% | 2.0% | 10.2% |
|  | ATRL | 0.0850 | 0.0014 | 1.1337 | 16.2% | 1.4% | 6.5% |
|  | SLFBR | 0.0800 | 0.0026 | 0.8636 | 11.5% | 5.2% | 4.5% |
|  | CSTL | 0.0750 | 0.0028 | 0.9618 | 12.2% | 14.4% | 14.5% |
|  | ILFR | 0.0640 | 0.0100 | 0.8751 | 15.3% | 7.7% | 10.0% |
|  | SLFBL | 0.0650 | 0.0110 | 0.8688 | 9.4% | 7.3% | 6.5% |
| **Male** | | | | | | | |
| **Metric** | **Fiber Tract** | **R2** | ***p-*value** | **FBP- FTP-  (n = 105)** | **FBP+ FTP-  (n = 71)** | **FBP- FTP+  (n = 1)** | **FBP+ FTP+  (n = 23)** |
| Fractional  Anisotropy | CgLR | 0.0680 | 0.0014 | 0.2713 | -10.0% | -0.5% | -20.6% |
|  | CSTR | 0.0520 | 0.0100 | 0.4124 | -7.5% | -8.6% | -6.4% |

**Table S4. Correlation between DTI metrics and MMSE: Females vs Males (*p* < .05 only)**

| **Female** | | | | | | | |
| --- | --- | --- | --- | --- | --- | --- | --- |
| **Metric** | **Fiber Tract** | **ß-Coefficient** | **CI low** | **CI high** | ***p-*value** | **R2** | **Adjusted R2** |
| Fractional Anisotropy | CCO | 6.8301 | 3.4212 | 10.2389 | 0.0001 | 0.1487 | 0.1194 |
|  | IFOR | 7.6824 | 3.6587 | 11.7061 | 0.0002 | 0.1422 | 0.1126 |
|  | CSTL | 7.5530 | 3.5057 | 11.6003 | 0.0003 | 0.1393 | 0.1096 |
|  | IFOL | 7.5640 | 3.3570 | 11.7710 | 0.0005 | 0.1348 | 0.1050 |
|  | CSTR | 7.3560 | 3.2169 | 11.4951 | 0.0006 | 0.1335 | 0.1036 |
|  | ILFR | 7.4528 | 3.2389 | 11.6667 | 0.0006 | 0.1329 | 0.1030 |
|  | ILFL | 7.3882 | 2.9539 | 11.8226 | 0.0012 | 0.1265 | 0.0964 |
|  | ATRL | 5.8245 | 1.5458 | 10.1031 | 0.0079 | 0.1092 | 0.0785 |
|  | UNCL | 5.6612 | 1.2905 | 10.0319 | 0.0114 | 0.1058 | 0.0750 |
|  | ATRR | 5.6421 | 1.0006 | 10.2836 | 0.0175 | 0.1019 | 0.0710 |
|  | SLFBR | 4.8544 | 0.8182 | 8.8906 | 0.0187 | 0.1013 | 0.0703 |
|  | UNCR | 5.1162 | 0.7592 | 9.4733 | 0.0216 | 0.1000 | 0.0690 |
|  | CgUL | 3.6184 | 0.5291 | 6.7077 | 0.0220 | 0.0999 | 0.0688 |
|  | CCF | 5.1132 | 0.4826 | 9.7438 | 0.0306 | 0.0969 | 0.0657 |
|  | CgLR | 4.0622 | 0.0493 | 8.0751 | 0.0473 | 0.0930 | 0.0618 |
| Mean Diffusivity | CSTL | -3.1510 | -4.4902 | -1.8118 | 0.0000 | 0.1913 | 0.1610 |
|  | ATRL | -1.9466 | -3.1038 | -0.7895 | 0.0011 | 0.1443 | 0.1112 |
|  | IFOL | -1.3757 | -2.2048 | -0.5466 | 0.0013 | 0.1475 | 0.1159 |
|  | ILFR | -1.9808 | -3.2548 | -0.7069 | 0.0025 | 0.1272 | 0.0951 |
|  | ILFL | -1.5739 | -2.6069 | -0.5408 | 0.0030 | 0.1401 | 0.1085 |
|  | CCO | -1.6617 | -2.7611 | -0.5623 | 0.0033 | 0.1334 | 0.1000 |
|  | CgLR | -1.3601 | -2.3620 | -0.3581 | 0.0081 | 0.1289 | 0.0969 |
|  | SLFBL | -2.6726 | -4.7300 | -0.6153 | 0.0112 | 0.1081 | 0.0746 |
|  | UNCR | -1.2019 | -2.1372 | -0.2667 | 0.0121 | 0.1156 | 0.0833 |
|  | CCF | -2.0016 | -3.5853 | -0.4180 | 0.0136 | 0.1205 | 0.0865 |
|  | CgUL | -1.6043 | -2.8906 | -0.3181 | 0.0148 | 0.1247 | 0.0923 |
|  | SLFBR | -2.4577 | -4.4369 | -0.4785 | 0.0153 | 0.1129 | 0.0798 |
|  | CgLL | -0.5210 | -1.0180 | -0.0241 | 0.0400 | 0.1194 | 0.0870 |
| **Male** | | | | | | | |
| **Metric** | **Fiber Tract** | **ß-Coefficient** | **CI low** | **CI high** | ***p-*value** | **R2** | **Adjusted R2** |
| Apparent Fiber Density | CgUR | -4.6969 | -8.8477 | -0.5461 | 0.0268 | 0.1130 | 0.0850 |
|  | CgUL | -4.0951 | -8.1103 | -0.0799 | 0.0457 | 0.1080 | 0.0800 |
| Complexity | CgUL | 3.1759 | 0.1161 | 6.2357 | 0.0420 | 0.1090 | 0.0810 |
| Mean Diffusivity | UNCL | -2.1797 | -3.9604 | -0.3989 | 0.0167 | 0.1160 | 0.0880 |

**Table S5. Correlation between DTI metrics and MoCA: Female vs Male (*p* < 0.05 only)**

| **Female** | | | | | | | |
| --- | --- | --- | --- | --- | --- | --- | --- |
| **Metric** | **Fiber Tract** | **ß-Coefficient** | **CI low** | **CI high** | ***p-*value** | **R2** | **Adjusted R2** |
| Fractional Anisotropy | CSTR | 19.4315 | 11.1642 | 27.6988 | 0.0000 | 0.1942 | 0.1662 |
|  | CSTL | 18.6856 | 10.5536 | 26.8176 | 0.0000 | 0.1902 | 0.1621 |
|  | ILFR | 19.1170 | 10.6677 | 27.5663 | 0.0000 | 0.1876 | 0.1594 |
|  | IFOR | 17.1921 | 9.0238 | 25.3605 | 0.0001 | 0.1761 | 0.1475 |
|  | CCO | 14.5101 | 7.5566 | 21.4635 | 0.0001 | 0.1748 | 0.1462 |
|  | ATRL | 17.3394 | 8.7886 | 25.8901 | 0.0001 | 0.1707 | 0.1420 |
|  | ILFL | 17.5079 | 8.5208 | 26.4949 | 0.0002 | 0.1653 | 0.1363 |
|  | UNCL | 16.7396 | 7.9789 | 25.5004 | 0.0002 | 0.1628 | 0.1337 |
|  | IFOL | 16.3506 | 7.7769 | 24.9243 | 0.0002 | 0.1625 | 0.1335 |
|  | CgLR | 15.2002 | 7.2062 | 23.1942 | 0.0002 | 0.1621 | 0.1331 |
|  | ATRR | 15.4591 | 6.0729 | 24.8454 | 0.0014 | 0.1461 | 0.1165 |
|  | CCF | 14.0666 | 4.6933 | 23.4399 | 0.0035 | 0.1377 | 0.1077 |
|  | UNCR | 13.1237 | 4.2844 | 21.9631 | 0.0038 | 0.1368 | 0.1068 |
|  | SLFBR | 11.9282 | 3.7273 | 20.1292 | 0.0046 | 0.1351 | 0.1051 |
|  | CgLL | 11.7465 | 3.3232 | 20.1698 | 0.0065 | 0.1319 | 0.1018 |
|  | SLFBL | 9.9252 | 0.7638 | 19.0866 | 0.0339 | 0.1173 | 0.0866 |
| Mean Diffusivity | ILFL | -4.9278 | -7.0429 | -2.8127 | 0.0000 | 0.2206 | 0.1918 |
|  | CgLR | -4.3470 | -6.4135 | -2.2805 | 0.0001 | 0.1997 | 0.1701 |
|  | IFOL | -2.8680 | -4.6120 | -1.1241 | 0.0014 | 0.1799 | 0.1493 |
|  | CgUL | -4.3005 | -6.9933 | -1.6077 | 0.0019 | 0.1705 | 0.1396 |
|  | ILFR | -3.9119 | -6.5645 | -1.2593 | 0.0041 | 0.1455 | 0.1138 |
|  | ATRL | -3.2757 | -5.7172 | -0.8342 | 0.0089 | 0.1558 | 0.1229 |
|  | CSTL | -3.7404 | -6.6471 | -0.8337 | 0.0120 | 0.1523 | 0.1203 |
|  | CCF | -4.1781 | -7.4815 | -0.8747 | 0.0135 | 0.1560 | 0.1231 |
|  | IFOR | -1.2693 | -2.4034 | -0.1352 | 0.0285 | 0.1539 | 0.1223 |
|  | CCO | -2.3854 | -4.7104 | -0.0605 | 0.0444 | 0.1420 | 0.1088 |
| **Male** | | | | | | | |
| **Metric** | **Fiber Tract** | **ß-Coefficient** | **CI low** | **CI high** | ***p-*value** | **R2** | **Adjusted R2** |
| Apparent Fiber Density | CgUL | -9.8266 | -17.8081 | -1.8452 | 0.0161 | 0.2050 | 0.1802 |
|  | CgUR | -10.0063 | -18.2827 | -1.7299 | 0.0181 | 0.2042 | 0.1793 |
|  | CSTR | -8.6493 | -16.1533 | -1.1453 | 0.0241 | 0.2021 | 0.1771 |
|  | CSTL | -8.2596 | -15.6698 | -0.8494 | 0.0291 | 0.2007 | 0.1757 |
| Fractional Anisotropy | CCO | 11.8559 | 3.2605 | 20.4513 | 0.0071 | 0.2110 | 0.1864 |
|  | IFOL | 10.8936 | 1.8547 | 19.9325 | 0.0184 | 0.2040 | 0.1791 |
|  | ATRL | 9.8037 | 1.4659 | 18.1414 | 0.0214 | 0.2029 | 0.1780 |
|  | ILFL | 10.1585 | 0.9393 | 19.3778 | 0.0310 | 0.2003 | 0.1753 |
| Mean Diffusivity | CgLR | -3.3776 | -5.7197 | -1.0356 | 0.0049 | 0.2050 | 0.1793 |
|  | CgLL | -2.0369 | -3.9156 | -0.1582 | 0.0337 | 0.1979 | 0.1722 |

**Table S6. List of acronyms**

| AD | Alzheimer's disease |
| --- | --- |
| ADNI | Alzheimer's disease neuroimaging initiative |
| AFD | Apparent fiber density |
| ANOVA | Analysis of Variance |
| CHARMED | Composite hindered and restricted model of diffusion |
| CI | 95% Confidence Interval |
| CX | Complexity |
| DTI | Diffusion tensor imaging |
| FA | Fractional anisotropy |
| FBP | Florbetapir |
| FN | Fixel number |
| FTP | Flortaucipir |
| GE | General Electric |
| MD | Mean diffusivity |
| MMSE | Mini-mental state examination |
| MoCA | Montreal cognitive assessment |
| MRI | Magnetic resonance imaging |
| NODDI | Neurite orientation dispersion and density imaging |
| PET | Positron emission tomography |
| SUVR | Standardized uptake value ratio |
